# Neuropathology in a diverse cohort of oldest‐old: The LifeAfter90 study

**DOI:** 10.1002/alz.71634

**Published:** 2026-07-24

**Authors:** Brittany N. Dugger, Melanie N. Luu, Viharkumar Patel, Charles DeCarli, Lee‐Way Jin, Paola Gilsanz, Dan Mungas, Claudia Kawas, Maria M. Corrada, Rachel A. Whitmer

**Affiliations:** ^1^ University of California Davis Sacramento California USA; ^2^ Kaiser Permanente Northern California, Division of Research Pleasanton California USA; ^3^ University of California, Irvine Irvine California USA

**Keywords:** aging, amyloid, cardiovascular disease, centenarian, cognitive, dementia, neuropathology, tau

## Abstract

**INTRODUCTION:**

Studies of the oldest‐old show great neuropathologic heterogeneity; little is known in diverse populations after age 90.

**METHODS:**

LifeAfter90 is a lifecourse cohort study of individuals aged ≥ 90 years evaluated every 6 months with optional brain donation; this study presents initial neuropathological findings.

**RESULTS:**

A total of 124 decedents (mean age 96, 49.2% White, 12.1% Black, 16.9% Asian, 18.5% Latino individuals) came to autopsy. At last evaluation, 35% had dementia, 23% cognitive impairment, and 41% normal cognition. 35.5% had intermediate AD, 8.1% had high AD neuropathologic changes, 73% had moderate/severe arteriolosclerosis, 23% one or more microinfarcts, 32% Lewy bodies, 24% TDP‐43 deposits, and 4% hippocampal sclerosis. There was a high degree of mixed neuropathology, with 69% having three or more pathologies. Cognitive impairment was most strongly associated with AD pathology.

**DISCUSSION:**

Multiple pathologies were common, and many individuals maintained normal cognition indicating substantial neuropathologic burden may be present in the absence of overt cognitive impairment, especially in the oldest‐old.

## Highlights

- Multiple neuropathologies were frequently found in those 90 years of age and older aligning with prior literature.
- Although many individuals contained multiple pathologies, most pathologies were at low stages/phases.
- Alzheimer disease pathologies were associated with cognitive impairment.

## 1 BACKGROUND

People aged 90 years or older are the fastest‐growing demographic segment in the United States, growing to approximately 2.6 million (0.77% of the total population) in 2022 and projected to reach 3.2 million (0.93% of the total population) by 2030.^1^ Alzheimer disease (AD) and related dementias (ADRD) incidence and prevalence has been shown to increase exponentially with age beginning at age 65; in a northern California, over a 6‐year period, 33% of individuals over 90 years of age were diagnosed with dementia.^2^ Prior studies in those over the age of 90 when compared to younger elderly have revealed differential frequencies of cognitive impairment and dementia risk factors, such as hypertension.^3^, ^4^, ^5^ Furthermore, many studies on the oldest‐old within the United States have been conducted predominately on non‐Hispanic White individuals resulting in a dearth of studies in cohorts with a range of ethnoracial backgrounds.^6^

Prior neuropathological work in those over the age of 90 has shown vascular and multiple pathologies are common and contribute to cognitive impairment as well as AD specific pathologies. Neuropathologic evaluations are tantamount in understanding etiological contributions to as they can reveal the full spectrum of underlying pathologies within an individual, such as TDP‐43 and Lewy body deposits, pathologies associated with cerebrovascular disease (arteriolosclerosis, cerebral amyloid angiopathy, and infarcts/hemorrhages), in addition to AD neuropathologic changes.^7^, ^8^, ^9^ There have been a handful of studies on the neuropathology of the oldest‐old revealing the multiple etiologies of cognitive impairment in very late life.^10^, ^11^, ^12^, ^13^, ^14^, ^15^ With respect to dementia in those over age 90, their brains can contain additional pathologies other than AD, including cerebrovascular, Lewy body, and TDP‐43 pathologies.^3^, ^10^, ^13^, ^16^, ^17^ Most of these studies are in individuals who identify as non‐Hispanic White; to date little is known on the neuropathological contributions to cognitive impairment in diverse individuals over the age of 90.^18^ It is important to have heterogeneous cohorts as they maximize variability in risk and protective factors as well as aid in the generalizability of findings to a wider population.^6^, ^18^ Although there have been great strides in neuropathologic cohort studies, there is a dearth of studies including individuals across heterogeneous demographics.^18^, ^19^, ^20^, ^21^, ^22^, ^23^, ^24^

The LifeAfter90 study was initiated in July 2018 with an overarching goal of advancing the understanding of lifecourse contributions to brain health and ADRD in individuals at least 90 years old. LifeAfter90 is an ongoing cohort study of Kaiser Permanente Northern California members examining cognitive aging, neuroimaging, and neuropathologic changes in individuals aged 90 years and older who come from heterogeneous demographic backgrounds. This study presents our initial neuropathologic findings as well as select clinical aspects, including cognitive impairment, of the first 124 participants who have come to autopsy.

**RESEARCH IN CONTEXT**

1. **Systematic review**: The literature was reviewed using standard sources (e.g., PubMed). While prior studies have examined neuropathology in individuals aged 90 and above these have been limited to persons of select demographics (such as studies consisting of persons who identify as non‐Hispanic White) and understanding neuropathologies within the oldest‐old among persons of various backgrounds is largely unexplored. This is critical to provide proper conclusions for all individuals aged 90 and above, regardless of their demographics.
2. **Interpretation**: In a heterogeneous cohort of individuals aged 90 and above, numerous pathologies were found. Pathologies associated with Alzheimer disease were associated with cognitive impairment. These findings are in alignment with prior studies, promoting the generalizability of knowledge to persons of more diverse backgrounds.
3. **Future directions**: Future research will involve a larger cohort (as many additional persons have enrolled) and should assess whether certain demographics may associate with neuropathologies within the oldest‐old. Additionally, examination of overall demographics in persons who enrolled in the study and those who did not, are warranted to understand potential barriers to research and autopsy involvement.

## 2 METHODS

### 2.1 Participants

LifeAfter90 eligible participants were English or Spanish speakers and members of Kaiser Permanente Northern California and at any point between 1964 and 2000 and were aged 90 or older at time of enrollment. Individuals were excluded from participation if at the time of initial enrollment in the study they had a diagnosis in their electronic medical records of any type of dementia or the presence of a health condition that would impede participation such as hospice, severe chronic obstructive pulmonary disease or congestive heart failure in the past 6 months, or end‐stage renal disease or dialysis in the past 12 months. Brain donation was available to all interested consenting participants. All participants provided consent during life and IRB for Kaiser Permanente Northern California and University of California Davis (UCD) approved protocols were used.

### 2.2 Examinations and cognitive diagnosis

Clinical exams including a cognitive assessment occur approximately every 6 months (i.e. biannually) from the time of enrollment until death following prior published methods.^25^ These study visits collected information on medical history, history of cognitive complaints and problems, physical function measures, physical and neurological exam, mental status testing using the Modified Mini‐Mental State Examination (3MS),^26^, ^27^, ^28^ Clinical Dementia Rating (CDR),^29^ and Functional Activities Questionnaire (FAQ)^30^ which has been modified to distinguish between different causes for functional impairment (cognitive vs other such as sensory or physical impairments).

Results of the clinical/cognitive assessment proximal to death were used to establish a clinical diagnosis (normal, cognitively impaired no dementia (CIND), dementia) using modified Diagnostic and Statistical Manual of Mental Disorders III‐R criteria.^31^ Normal cognition was diagnosed if there was no impaired cognitive domain (based on the 3MS) and there was no clinically significant functional impairment (based on CDR, FAQ, examining physician diagnosis). CIND was diagnosed if (a) there was one or more impaired cognitive domains and no clinically significant functional impairment, or (b) there was no impaired cognitive domain but there was clinically significant functional impairment. A diagnosis of dementia required one or more impaired cognitive domains and significant functional impairment.

### 2.3 Procurements and histological procedures

All procedures for procuring and preparing tissues were overseen by the UC Davis Neuropathology Core and followed best practice guidelines.^32^ The standard protocol for procurement included immersion fixation in 10% formalin of the left hemisphere, while the right hemisphere was coronally cut in ∼1 cm thick slice and immediately frozen upon slabs of dry ice. Hemi‐brains were immersion fixed in 10% formalin for a minimum of 21 days before gross dissection. After fixation, select neuroanatomic regions were sampled following guidelines set forth by the National Institute on Aging and Alzheimer's Association (NIA‐AA)^33^, ^34^ and then subjected to paraffin embedding, sectioning, and hematoxylin/eosin and immunohistochemical staining (see Table S1 for additional details). Areas sampled and subjected to hematoxylin and eosin staining included: superior frontal gyrus, superior and middle temporal gyri, pre and post central gyri, superior parietal lobule, visual cortex with Brodman area 17, posterior hippocampus at level of lateral geniculate nucleus, anterior and posterior cingulate cortices, amygdala, striatum at level of anterior commissure with nucleus basalis of Meynert, mammillary body with anterior diencephalons, midbrain with substantia nigra at the level of the third nerve, olfactory bulb and tract, cerebellar vermis (anterior), frontal, parietal, and occipital periventricular white matter, and anterior hippocampus. Table S1 includes details on immunohistochemical staining and stains for each region. Methodologies for sample preparation, cutting, and staining have been previously described and are consistent with other US based brain banks focused on ADRD.^35^ In brief, all staining was conducted at core facilities within UCD complying with all Federal, State of California, and UCD guidelines and regulations. Deviation of this protocol included the following: If abnormalities (such as stroke/hemorrhage) were found at the time of extraction‐ the affected hemisphere was immersion fixed and if the *post‐mortem* interval (PMI) was greater than 24 hours the whole brain was immersion‐fixed and no frozen samples were taken. Furthermore, gross abnormalities not indicative of neurodegenerative disease, e.g. large infarcts, neoplasm, etc., when present, were sampled during gross dissection, with subsequent processing and staining with hematoxylin and eosin reviewed microscopically and diagnosed.

#### 2.3.1 Pathological assessments and data collection

Three neurodegenerative pathology experts (B.D., L.W.J., V.P.) performed pathologic evaluations blinded to clinical diagnoses and demographic data. After initial review by each expert, consensus evaluations involving review of the pathologic diagnoses and accompanying slides and were conducted on all cases by the experts to decrease any intra‐rater variability. Assessments followed the guidelines outlined within the coding guidebooks of the National Alzheimer's Coordinating Center Neuropathology form versions 10 and 11, the NIA‐AA guidelines, Belvin *et al.*, Greenberg *et al*., BrainNET Europe Consortium for staging of neurofibrillary pathology, and dementia with Lewy bodies consortium.^33^, ^36^, ^37^, ^38^, ^39^, ^40^, ^41^, ^42^ All data were collected utilizing the National Alzheimer's Coordinating Center's neuropathology forms versions 10 or 11. Further descriptions are located below.

For AD pathologies, scoring of the density of AT8 immunohistochemically labeled neuropil threads across neuroanatomic regions (posterior/anterior hippocampus, temporal cortex, and occipital cortex) were used for staging of neurofibrillary pathology following BrainNET Europe Criteria.^42^ Thal amyloid phase was evaluated on 4G8 immunohistochemically stained tissues and determined by the presence of amyloid plaques (including diffuse) in anatomic locations outlined by Thal et al.^43^ and not lesion density. Neuritic plaque densities followed a modified CERAD criteria,^44^ modified referring to the use of AT8 immunohistochemistry to denote neuritic plaques.

The presence of aging‐related tau astrogliopathy (ARTAG)^45^ was evaluated in all AT8 immunohistochemistry‐stained areas (see Table S1), and was denoted as presence for the case if at least one area contained ARTAG.

Assessment of Lewy body disease pathologies (i.e. Lewy bodies [LBs] and Lewy neurites) was completed using LB509 immunohistochemically stained tissues, utilized the four stages as defined by the dementia with Lewy bodies consortium (0  =  absent, 1  =  mild–sparse LBs or LNs, 2  =  moderate–more than one LB per high power field and sparse LNs, 3  =  severe–more than four LBs and scattered LNs in low power field, 4  =  very severe–numerous LBs and LNs) and then following the McKeith 2017 criteria to report the Lewy body stage.^39^

Cerebral amyloid angiopathy was assessed on 4G8 immuohistochemically stained tissues using a modified NACC scoring system^24^ and denoted as the following: 0  =  none–absent, 1  =  mild–scattered positivity in parenchymal and/or leptomeningeal vessel in focal areas, 2  =  moderate–intense positivity in many parenchymal and/or leptomeningeal vessels, 3  =  severe–widespread (throughout the brain) intense vessel positivity. The modifications refer to the use of amyloid‐β (4G8) staining. For arteriolosclerosis, we followed guidelines by Belvin et al. semi‐quantitative assessments and based on the appearance of the majority of vessels within the frontal, parietal, and occipital periventricular white matter (0  =  none–normal, 1  =  mild–mild thickening of vessel medial, mild fibrosis, 2  =  moderate–partial loss of smooth muscle cells in the media, moderate hyaline fibrosis, 3  =  severe–complete loss of smooth muscle cells in media, severe hyaline fibrosis, lumen stenosis).^36^ For infarct results, only the presence or absence of old infarcts were considered (NACC variables of npold for microinfarcts and npinf for infarcts observed grossly, including lacunes). The presence of hippocampal sclerosis was defined by gliosis and pyramidal cell loss in CA1 and Subiculum of the posterior hippocampal formation out of proportion to AD neuropathologic change in the same region.^33^

For evaluation of TDP‐43 deposits, the presence of dystrophic neurites, neuronal cytoplasmic inclusions, and intranuclear inclusions were evaluated on posterior hippocampal, amygdaloid complex, inferior temporal, and superior frontal cortices. LATE‐NC Stages were based on criteria from prior publications^46^, ^47^; a case was considered Stage 1 if one of the following regions contained TDP‐43 deposits: amygdaloid complex, hippocampus, entorhinal cortex, or inferior temporal gyrus, for Stage 2 TDP‐43 deposits were found in both the amygdaloid complex and hippocampus, and for Stage 3 in all of the following regions amygdaloid complex, hippocampus, and superior frontal cortex.

### 2.4 Statistical analyses

We examined participant characteristics by cognitive status at last clinical evaluation before death. We report numbers and proportions for categorical variables and means and standard deviations for continuous variables. We used Fisher's exact test to compare the proportions of categorical variables and analysis of variance (ANOVA) to compare means of continuous variables across cognitive groups. The relationship between neuropathological findings and cognitive status at last evaluation was examined in 124 participants who underwent autopsy and have completed neuropathologic evaluations. All neuropathological variables were analyzed as categorical predictors. We conducted two sets of analyses examining different cognitive outcomes: (1) dementia vs no dementia (combining normal cognition and CIND) and (2) cognitive impairment (combining CIND and dementia) vs normal. For each outcome, we performed separate binary logistic regressions with each categorical neuropathological variable as the predictor. We implemented Firth's penalized maximum binary logistic regression^48^ due to its ability to address potential biases due to small numbers and complete separation in the data (where certain pathology categories perfectly predicted the outcome). For each categorical neuropathological predictor, we report the Type 3 Analysis of Effects to test the overall significance of the variable as well as odd ratios and 95% confidence intervals for each comparison. Also, because of small numbers, we combined some categories for analysis: for B‐score we combined Scores 0 and 1 (i.e. Tau stages 0‐II), and for atherosclerosis we combined none and mild. All models were adjusted only for age at death, because sex, race/ethnicity, education, and interval between last evaluation and death were not associated with either outcome of dementia or cognitive impairment. All analyses were done using SAS 9.4 statistical software (SAS Institute Inc., Cary, NC).

## 3 RESULTS

### 3.1 Participant demographics

As of January 2025, 1,134 individuals were enrolled in the LifeAfter90 study (25% Asian, 23% Black/African American, 20% Latino, 7% multi/other, and 25% White); 444 (39.5%) individuals died, regardless of autopsy enrollment. Furthermore, of the 1,134 individuals, enrolled 390 (34.4%) enrolled in the autopsy program (22% Asian, 19.5% Black/African American, 17.5% Latino, 8.5% Multiracial/Other, and 32.5% White). Of the 390 participants consented to autopsy, 155 (39.7%) had died, with autopsies completed in 124 (80%). A total of 31 consented individuals died without undergoing an autopsy: seven identified as Asian, eight as Black/African American, five as Latino, eight as White, and two as Other.

The final analysis included 124 individuals who had died and had completed neuropathological evaluations (17.4% Asian, 12.4% Black/African American, 19% Latino, 0.8% multi/other, and 50.4% White). The mean age of death was 95.3 years (range 90.3‐105.5) and 75 (60.5%) were women. With respect to education, nearly three quarters of participants had some college or higher (74.6%). At last evaluation, the mean 3MS Score, a modified mini‐mental state examination that measures cognitive function, was 80.2 (range 30.0–100.0). At autopsy, brain weight averaged 1,158 grams (range 875–1455 grams). See Table 1 totals for additional details.

**TABLE 1:** Characteristics of participants by cognitive status at last evaluation.

Characteristic	Normal (*N* = 52)	CIND (*N* = 29)	Dementia (*N* = 43)	Total (*N* = 124)	*p*‐value^a^
	***N* (%)**				
Sex					
Female	33 (63.5%)	17 (58.6%)	25 (58.1%)	75 (60.5%)	0.85
Male	19 (36.5%)	12 (41.4%)	18 (41.9%)	49 (39.5%)	
Race/ethnicity					
Asian	7 (13.5%)	6 (20.7%)	8 (18.6%)	21 (16.9%)	0.88
Black	7 (13.5%)	3 (10.3%)	5 (11.6%)	15 (12.1%)	
Latino	9 (17.3%)	5 (17.2%)	9 (20.9%)	23 (18.5%)	
Multiple/Other/missing	2 (3.8%)	2 (6.9%)	0 (0.0%)	4 (3.2%)	
White	27 (51.9%)	13 (44.8%)	21 (48.8%)	61 (49.2%)	
Education					
<High School / GED	10 (19.6%)	07 (24.1%)	14 (33.3%)	31 (25.4%)	0.17
Some College/Trade	19 (37.3%)	11 (37.9%)	11 (26.2%)	41 (33.6%)	
Assoc. Degree / College	05 (09.8%)	06 (20.7%)	10 (23.8%)	21 (17.2%)	
Graduate School	17 (33.3%)	05 (17.2%)	07 (16.7%)	29 (23.8%)	
Missing	01 (01.9%)	00 (00.0%)	01 (02.3%)	02 (01.6%)	
	**Mean (range)**				
Age at death, years	95.4 (91.1–100.6)	95.3 (91.8–102.6)	96.8 (90.5–105.7)	95.9 (90.5–105.7)	0.021
Last 3MS score	92.1 (70.0–100.0)	82.1 (61.0–95.0)	63.8 (30.0–90.0)	80.2 (30.0–100.0)	<0.001
Time from last 3MS score to death, years	0.6 (0.1–3.5)	0.5 (0.0–1.6)	0.6 (0.0–2.2)	0.6 (0.0–3.5)	0.67
Brain weight, kg	1169 (937–1455)	1176 (892–1445)	1134 (875–1442)	1158 (875–1455)	0.20

Abbreviations: 3MS, Modified Mini‐Mental State Examination; CIND, cognitively impaired no dementia; GED, general education development.

^a^ *p*‐value from Fisher's exact test for categorical variables and ANOVA for continuous variables.

### 3.2 Pathologic findings

**FIGURE 1:**
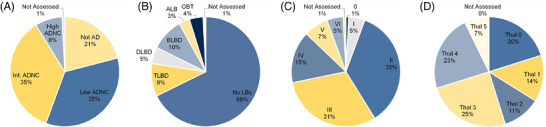
Frequencies of (A) NIA‐AA ADNC, (B) Consortium for DLB criteria, (C) BrainNet Europe Tau Stage, and (D) Thal amyloid phases of LifeAfter90 cases (*N* = 124). One participant showed no evidence of NFTs and 25 had no evidence of amyloid‐β plaques. ADNC, Alzheimer's disease neuropathologic changes; ALB, amygdala predominant Lewy bodies; BLBD, brainstem Lewy body disease; LBs, Lewy bodies; NIA/AA, National Institute on Aging and Alzheimer's Association; OBT,  olfactory bulb and tract only LBs; TLBD, transitional Lewy body disease.

**FIGURE 2:**
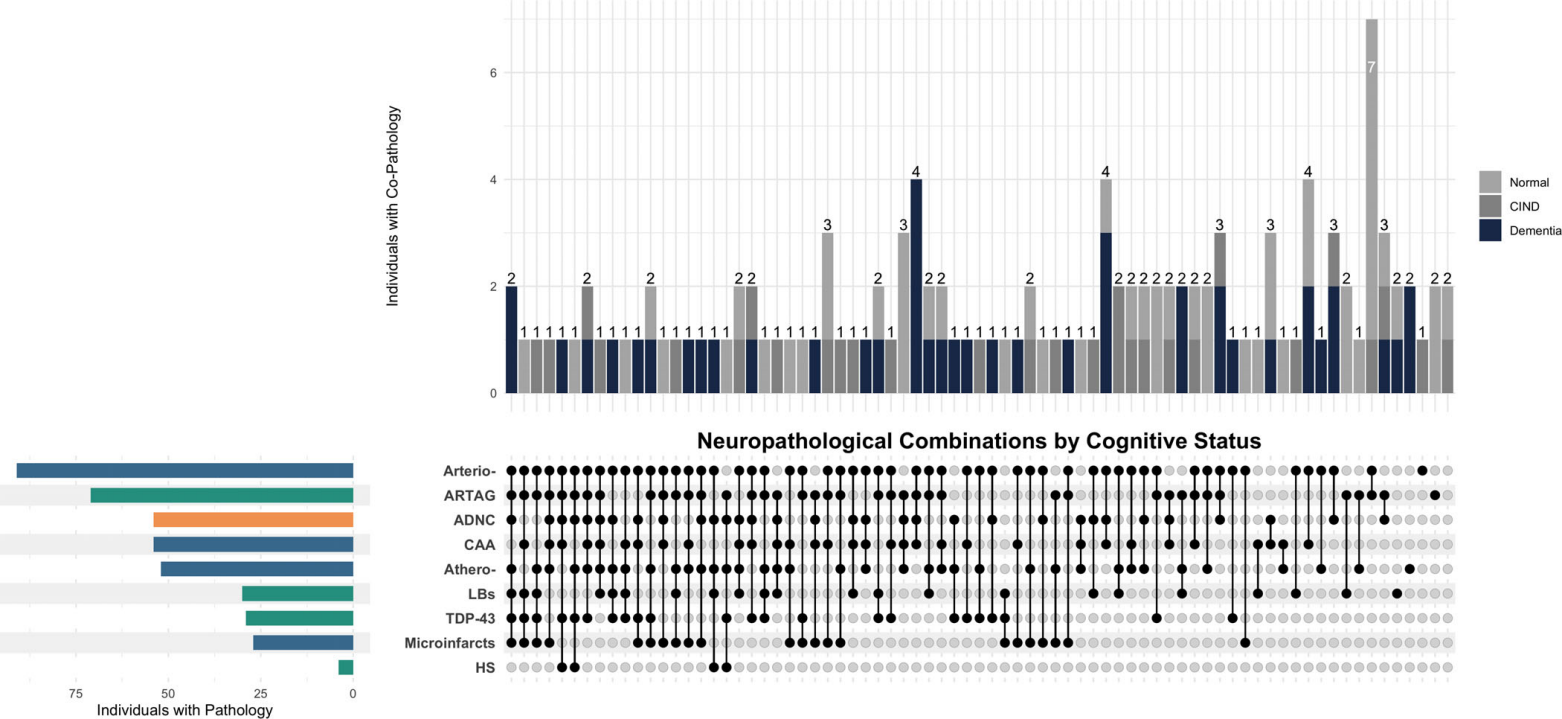
Upset plot depicting the burden of mixed pathologies by cognitive status. The bar chart in lower left core shows frequencies of individual neuropathologies. Connected black dots on the x‐axis indicate specific combinations of neuropathologies. Histograms in the main panel shows frequencies of the specific neuropathology combinations (color indicates cognitive status ‐ light grey Normal, dark grey CIND, blue Dementia). The height of each bar corresponds to the number of persons with each combination. Pathologies include: microinfarcts, hippocampal sclerosis, ARTAG, TDP‐43, arteriolosclerosis, atherosclerosis, CAA, LBs, and ADNC. CAA, Athero, and Arterio included moderate/frequent scores, ADNC included intermediate/high, and LBs included BLBD, TLBD, or DLBD diagnoses. TDP‐43 and ARTAG were based on presence, and microinfarcts included only old microinfarcts. ADNC, Alzheimer disease neuropathologic change; ARTAG, aging‐related tau astrogliopathy; BLBD, brainstem Lewy body disease; CAA, cerebral amyloid angiopathy; CIND, Cognitively impaired no dementia, LBs, Lewy bodies; TLBD, transitional Lewy body disease.

Details regarding the neuropathology of the entire cohort of 124 individuals are shown in Table 2, and Figures 1 and 2. AD and vascular pathologies were the most frequent findings. Although AD pathologies were present, most cases fell into the low (35%) or intermediate (35%) AD neuropathologic changes (ADNC) categories. Notably, 21% of participants were classified as Not ADNC. High ADNC was found in 10 participants (8%) (Figure 1A). For Tau stage (Figure 1C), the most frequent Tau stages were Stage II (35%) or Stage III (31%); advanced stages were less frequent. Only one participant showed no evidence of neuronal tau pathology (Stage 0). The most common Thal amyloid phases were Thal 3 (25%) and Thal 4 (23%), with advanced amyloid phase (Thal 5) being the least frequent (7%) (Figure 1D); 20.2% of the cohort did not have evidence of amyloid plaques (Thal Phase 0).

**TABLE 2:** Neuropathological features by cognitive status at last evaluation.

Parameter	Normal(*N* = 52)	CIND(*N* = 29)	Dementia(*N* = 43)	Total(*N* = 124)	*p*‐value^a^
ADNC					
None	13 (25.0%)	5 (17.2%)	8 (18.6%)	26 (21.0%)	0.012
Low	24 (46.2%)	11 (37.9%)	9 (20.9%)	44 (35.5%)	
Intermediate	15 (28.8%)	10 (34.5%)	19 (44.2%)	44 (35.5%)	
High	0 (0.0%)	3 (10.3%)	7 (16.3%)	10 (8.1%)	
Thal phase (A‐Score)					
0 (A0)	12 (23.1%)	5 (17.2%)	8 (18.6%)	25 (20.2%)	Thal phase 0.033
1 (A1)	12 (23.1%)	4 (13.8%)	1 (2.3%)	17 (13.7%)	A‐Score 0.042
2 (A1)	6 (11.5%)	5 (17.2%)	3 (7.0%)	14 (11.3%)	
3 (A2)	13 (25.0%)	6 (20.7%)	12 (27.9%)	31 (25.0%)	
4 (A3)	9 (17.3%)	7 (24.1%)	12 (27.9%)	28 (22.6%)	
5 (A3)	0 (0.0%)	2 (6.9%)	7 (16.3%)	9 (7.3%)	
Tau stage (B‐Score)					
0 (B0)	1 (1.9%)	0 (0.0%)	0 (0.0%)	1 (0.8%)	Tau stage 0.004
I (B1)	3 (5.8%)	1 (3.4%)	2 (4.7%)	6 (4.8%)	B‐Score^b^ 0.003
II (B1)	25 (48.1%)	10 (34.5%)	10 (23.3%)	45 (36.3%)	
III (B2)	20 (38.5%)	9 (31.0%)	9 (20.9%)	38 (30.6%)	
IV (B2)	3 (5.8%)	4 (13.8%)	12 (27.9%)	19 (15.3%)	
V (B3)	0 (0.0%)	3 (10.3%)	6 (14.0%)	9 (7.3%)	
VI (B3)	0 (0.0%)	2 (6.9%)	4 (9.3%)	6 (4.8%)	
CERAD neuritic plaque score (C Score)					
None (C0)	23 (44.2%)	9 (31.0%)	9 (20.9%)	41 (33.1%)	CERAD/C‐Score 0.005
Sparse (C1)	18 (34.6%)	14 (48.3%)	10 (23.3%)	42 (33.9%)	
Moderate (C2)	9 (17.3%)	3 (10.3%)	18 (41.9%)	30 (24.2%)	
Frequent (C3)	2 (3.8%)	3 (10.3%)	6 (14.0%)	11 (8.9%)	
Lewy body disease					
None/amygdala/olfactory	41 (78.8%)	19 (65.5%)	34 (79.1%)	94 (75.8%)	0.57
Brainstem	5 (9.6%)	5 (17.2%)	2 (4.7%)	12 (9.7%)	
Limbic	4 (7.7%)	4 (13.8%)	4 (9.3%)	12 (9.7%)	
Neocortical	2 (3.8%)	1 (3.4%)	3 (7.0%)	6 (4.8%)	
Hippocampal sclerosis					
Absent	49 (96.1%)	28 (100.0%)	40 (95.2%)	117 (96.7%)	0.68
Present	2 (3.9%)	0 (0.0%)	2 (4.8%)	4 (3.3%)	
Missing	1 (002.4%)	1 (000.0%)	1 (004.0%)	3 (002.2%)	
LATE‐NC					
None	39 (78.0%)	23 (82.1%)	29 (69.0%)	91 (75.8%)	0.72
Stage 1	4 (8.0%)	3 (10.7%)	3 (7.1%)	10 (8.3%)	
Stage 2	6 (12.0%)	2 (7.1%)	8 (19.0%)	16 (13.3%)	
Stage 3	1 (2.0%)	0 (0.0%)	2 (4.8%)	3 (2.5%)	
Missing	2	1	1	4	
ARTAG					
Absent	19 (36.5%)	13 (44.8%)	21 (48.8%)	53 (42.7%)	0.47
Present	33 (63.5%)	16 (55.2%)	22 (51.2%)	71 (57.3%)	
Old microinfarcts					
None	40 (78.4%)	21 (75.0%)	34 (79.1%)	95 (77.9%)	0.91
Present	11 (21.6%)	7 (25.0%)	9 (20.9%)	27 (22.1%)	
Missing	01 (002.4%)	01 (004.2%)	00 (000.0%)	02 (002.2%)	
Old infarcts observed grossly, including lacunes					
None	45 (86.5%)	23 (79.3%)	32 (74.4%)	100 (80.6%)	0.32
Present	7 (13.5%)	6 (20.7%)	11 (25.6%)	24 (19.4%)	
Cerebral amyloid angiopathy					
None	20 (38.5%)	12 (41.4%)	11 (25.6%)	43 (34.7%)	0.77
Mild	10 (19.2%)	5 (17.2%)	12 (27.9%)	27 (21.8%)	
Moderate	9 (17.3%)	5 (17.2%)	10 (23.3%)	24 (19.4%)	
Severe	13 (25.0%)	7 (24.1%)	10 (23.3%)	30 (24.2%)	
Atherosclerosis					
None	8 (15.4%)	0 (0.0%)	3 (7.3%)	11 (9.1%)	0.15
Mild	24 (46.2%)	14 (50.0%)	21 (51.2%)	59 (48.8%)	
Moderate	18 (34.6%)	9 (32.1%)	12 (29.3%)	39 (32.5%)	
Severe	2 (3.8%)	5 (17.9%)	5 (12.2%)	12 (9.9%)	
Missing	00 (002.4%)	01 (004.2%)	02 (000.0%)	03 (002.2%)	
Arteriolosclerosis					
None	1 (1.9%)	0 (0.0%)	0 (0.0%)	1 (0.8%)	0.48
Mild	18 (34.6%)	5 (17.2%)	9 (20.9%)	32 (25.8%)	
Moderate	26 (50.0%)	19 (65.5%)	27 (62.8%)	72 (58.1%)	
Severe	7 (13.5%)	5 (17.2%)	7 (16.3%)	19 (15.3%)	
White matter rarefaction					
None	6 (11.5%)	0 (0.0%)	2 (4.7%)	8 (6.5%)	0.32
Mild	22 (42.3%)	13 (44.8%)	14 (32.6%)	49 (39.5%)	
Moderate	13 (25.0%)	11 (37.9%)	14 (32.6%)	38 (30.64%)	
Severe	11 (21.2%)	5 (17.2%)	13 (30.2%)	29 (23.4%)	

Missing data: MMSE *n* = 1 normal, *n* = 3 dementia; brain weight *n* = 1 normal, *n* = 1 CIND.

Abbreviations: ADNC, Alzheimer's disease neuropathologic change; ARTAG, aging‐related tau astrogliopathy; CIND, cognitively impaired no dementia; LATE‐NC, limbic associated TDP‐43 encephalopathy neuropathologic change; LB, Lewy bodies; MMSE, Mini‐Mental State Examination; WMR, white matter rarefaction

^a^ *p*‐value from Fisher's exact test.

^b^ For B‐score, B0 and B1 were combined to allow for calculation.

With respect to Lewy pathologies, most cases revealed no evidence of LBs and/or Lewy neurites (68%); among the cases with Lewy pathology (31%), the highest frequencies were with transitional/limbic (TLBD, 9.7%) and brainstem predominant (BLBD, 9.7%) subtypes. Other classifications, including diffuse/neocortical LB disease (DLBD, 5%), olfactory bulb type (OBT, 4%), and amygdala‐predominant (ALB, 3%) (Figure 1B).

Frequency/distribution/severity of other neuropathologies in the cohort, including microinfarcts, hippocampal sclerosis (HS), ARTAG, cerebral amyloid angiopathy (CAA), atherosclerosis, arteriolosclerosis, and TDP‐43 deposits are located within Table 2. Microinfarcts were predominantly absent in most participants (∼80%). For HS, most cases (97%) showed no evidence of HS with only four having HS. ARTAG, on the other hand, was present in 85% of cases. For CAA, a global gradient of severity was observed, distributed across none (34.7%), mild (21.8%), moderate (19.4%), and severe (24.2%) classifications (Table 2). Similar patterns were noted for atherosclerosis and arteriolosclerosis, although atherosclerosis appeared to have a lower prevalence of severe pathology compared to arteriolosclerosis. TDP‐43 presence was categorized based on the presence of neuronal cytoplasmic inclusions, dystrophic neurites, and/or neuronal intranuclear inclusions in select brain regions, including the superior frontal gyrus, posterior hippocampus, entorhinal cortex/inferior temporal region, and amygdaloid complex, with 23.8% of the cohort displaying TDP‐43 pathology in one or more of these regions.

For cases with TDP‐43 deposits, all cases had some degree of AD tau and/or amyloid deposits (i.e Thal phase and/or Tau stage not 0); 10 cases (∼34%) had concomitant TDP‐43 and Lewy pathologies. Three out of the four HS cases had TDP‐43 deposits. With respect to overlap of LB pathology and ADNC, 31 out of 39 cases with Lewy pathologies also had at least Low ADNC.

To evaluate pathologic heterogeneity, we examined frequencies of nine total pathologies defining them as follows: CAA, atherosclerosis, and arteriolosclerosis included only those with moderate/frequent severity, ADNC included only ADNC intermediate/high, and LBs included a diagnosis of BLBD, TLBD, or DLBD; TDP‐43 and ARTAG were based on presence in any anatomic area, and microinfarcts included only old microinfarcts. The presence of these pathologies by cognitive status are illustrated in Figure 2; revealing a very heterogenous array‐ both among individuals and across cognitive status.

For additional clinicopathological defined diseases recorded within the NACC forms, one case with dementia met criteria for progressive supranuclear palsy.^49^ There were five cases (4% of the cohort) with brain tumor diagnoses. One case had evidence of metastatic non‐small cell adenocarcinoma with hemorrhages. Another had evidence of metastatic neoplasm involving cerebellum and cerebellar leptomeninges. Three of the five tumor cases had evidence of meningiomas (2% of the entire 124 cohort)‐ of these three‐ one case had incidental meningioma, World Health Organization (WHO) grade 1, transitional type, one with Meningioma‐CNS WHO grade 1, and one with a dural mass proximal to left orbital frontal gyrus with gross features suspicious for meningioma. No cases had pathological or clinical evidence of corticobasal degeneration, chronic traumatic encephalopathy, Creutzfeldt‐Jacob disease, multiple systems atrophy, Pick's disease, or frontotemporal lobar degeneration.

### 3.3 Relation of neuropathology finds to cognitive status

Cognitive status at the last assessment was classified as dementia (*n* = 43, 35%), CIND (*n* = 29, 23%), and normal cognition (*n* = 52, 42%). Among participants classified as having high ADNC (*n* = 10, 8%) the majority (70%) were diagnosed with dementia (Table 2). In contrast, individuals with no ADNC (*n* = 26, 21%) were predominantly diagnosed as either cognitively normal or CIND, with half being cognitively normal. Low and intermediate ADNC groups showed a gradual increase in cognitive impairment, with dementia present in 20% and 43% of cases, respectively. Hence, the distribution of Thal amyloid phase/A score and Tau stage/B score, and CERAD neuritic plaques/C score (which make up ADNC), varied by cognitive status (Table 2). In contrast, the distribution of vascular pathologies, including infarcts, cerebral amyloid angiopathy, arteriolosclerosis, and atherosclerosis did not vary by cognitive status. This was also true for Lewy body pathology‐ although numbers were low in each group. Among those with TDP‐43 inclusions, 62% (*n* = 18) had some degree of cognitive impairment, with 45% (*n* = 13) evaluated as having dementia, although groups were not significantly different. HS was rare (*n* = 4, 3%) with 2/4 of the HS cases having dementia at last assessment before death.

Results of analyses relating neuropathological findings to dementia or to cognitive impairment are shown in Table 3. For dementia as the outcome, the Type 3 analysis revealed significant overall effects for two of the individual AD scores (C‐score and B‐score) with the odds of dementia after adjusting for age at death (C‐Score: *χ* ^2^ = 9.80, df = 3, *p* = 0.020; B‐Score: χ^2^ = 6.71, df = 2, *p* = 0.035). Compared to people with a C‐score of no pathology, those with moderate pathology had increased odds of dementia (odds ratio [OR] = 4.41, 95% confidence interval [CI] = 1.61–12.80, *p* = 0.0055) while C‐Scores of sparse neuritic plaque pathology or frequent neuritic plaque pathology showed elevated but non‐significant associations. Similarly, B‐Score demonstrated that the highest AD tau pathology level (Score 3 vs 0–1) was associated with more than five times the odds of dementia (OR = 5.17, 95% CI = 1.5–18.92, *p* = 0.011). A‐Score showed a significant overall effect (χ^2^ = 8.37, df = 3, *p* = 0.039), though individual category comparisons did not reach statistical significance. ADNC approached significance (χ^2^ = 6.42, df = 3, *p* = 0.093), with the highest category showing a trend toward increased odds of dementia. None of the other neuropathological variables showed significant associations with dementia, although some cells have low numbers.

**TABLE 3:** Association of neuropathology with dementia and cognitive impairment.*

	Dementia			Cognitive impairment		
Parameter	OR	95% CI	Type III *p*‐value	OR	95% CI	Type III *p*‐value
**ADNC**						
None	1.00	ref		1.00		
Low	0.64	0.21–1.95	0.093	0.87	0.33–2.29	0.097
Intermediate	1.57	0.58–4.49		1.85	0.70–4.95	
High	3.84	0.86–19.91		18.94	2.04–> 999.99	
**A‐Score**						
0	1.00	ref		1.00		
1 (Thal phase 1/2)	0.35	0.09–1.26	0.039	0.70	0.24–1.99	0.077
2 (Thal phase 3)	1.27	0.42–3.92		1.23	0.43–3.54	
3 (Thal phase 4/5)	2.08	0.73–6.22		2.69	0.94–8.03	
**B‐Score**						
0‐1 (Tau stage 0‐II)	1.00	ref		1.00		
2 (Tau stage III/IV)	1.91	0.83–4.53	0.035	1.83	0.86–3.93	0.028
3 (Tau stage V/IV)	5.17	1.54–18.92		35.02	4.27–> 999.99	
**C‐Score**						
None	1.00	ref		1.00		0.141
CERAD Sparse	1.23	0.44–3.46	0.020	1.79	0.76–4.33	
CERAD Moderate	4.41	1.61–12.8		2.61	0.99–7.22	
CERAD Frequent	3.36	0.84–13.82		4.28	1.04–24.69	
**HS**						
Absent	1.00	ref		1.00		
Present	1.77	0.25–12.26	0.583	0.67	0.10–4.51	0.692
**LATE‐NC**						
None	1.00	ref		1.00		
Stage 1	0.94	0.20–3.62	0.584	1.07	0.30–4.19	0.996
Stage 2	1.64	0.54–4.90		1.01	0.34–3.14	
Stage 3	3.92	0.49–44.78		1.32	0.17–14.84	
**LB**						
None/Amygdala/ Olfactory	1.00	ref		1.00		
Brainstem	0.44	0.08–1.67	0.664	1.11	0.34–3.81	0.951
Limbic	0.83	0.22–2.81		1.39	0.42–5.15	
Neocortical	1.64	0.29–8.85		1.32	0.27–8.04	
**ARTAG**						
Absent	1.00	ref		1.00		
Present	0.75	0.35–1.6	0.455	0.68	0.32–1.40	0.302
**Micro‐infarcts**						
None	1.00	ref		1.00		
Present	0.88	0.34–2.15	0.786	1.03	0.44–2.49	0.948
**Infarcts**						
None	1.00	ref		1.00		
Present	1.60	0.62–4.04	0.331	1.80	0.71–4.88	0.236
**CAA**						
None	1.00	ref		1.00		
Mild	1.93	0.68–5.53	0.371	1.30	0.49–3.53	0.843
Moderate	2.51	0.85–7.52		1.57	0.57–4.43	
Severe	1.39	0.49–3.91		1.10	0.43–2.81	
**Athero‐sclerosis**						
None	1.00	ref		1.00		
Mild	1.57	0.42–7.14	0.902	3.86	1.05–17.32	0.125
Moderate	1.24	0.31–5.89		3.04	0.79–14.05	
Severe	1.57	0.29–9.38		9.73	1.69–77.11	
**Arteriolo‐sclerosis**						
None/mild	1.00	ref		1.00		
Moderate	1.31	0.54–3.36	0.754	2.18	0.95–5.10	0.167
Severe	1.56	0.46–5.19		2.26	0.74–7.32	
**WMR**						
None	1.00	ref		1.00		
Mild	0.89	0.19–5.44	0.558	2.95	0.67–17.4	0.315
Moderate	1.39	0.30–8.49		4.65	1.02–28.33	
Severe	1.78	0.37–11.24		3.80	0.80–23.78	

Abbreviations: ADNC, Alzheimer's disease neuropathologic change; ARTAG, aging‐related tau astrogliopathy; CAA, cerebral amyloid angiopathy; CI, confidence interval; HS, hippocampal sclerosis; LATE‐NC, limbic associated TDP‐43 encephalopathy neuropathologic change; LB, Lewy bodies; OR, odds ratio; WMR, white matter rarefaction.

* Odds ratios and confidence intervals were estimated using Firth's logistic regression (see methods) adjusting for age at death. The *p*‐value corresponds to the type 3 analyses and tests the overall effect of the categorical neuropathological variable on the outcome.

In relation to cognitive impairment as the outcome, the Type 3 analysis revealed only one neuropathological variable with a significant overall effect. B‐Score showed a significant overall association with the odds of cognitive impairment (χ^2^ = 7.15, df = 2, *p* = 0.028), driven primarily by the highest AD tau pathology category (Score 3 (i.e. Tau stage V/VI) vs 0‐1 (Tau stage 0‐II): OR = 35.02, 95% CI = 4.27– > 999.99, *p* = 0.018). A‐Score approached significance (χ^2^ = 6.85, df = 3, *p* = 0.077), with the highest category showing elevated but non‐significant odds of cognitive impairment (Score 3 (Thal 4/5) vs 0 (Thal 0): OR = 2.69, 95% CI = 0.94–8.03, *p* = 0.075). ADNC also approached significance (χ^2^ = 6.32, df = 3, *p* = 0.097), though individual categories did not. C‐Score did not show a significant overall effect (χ^2^ = 5.47, df = 3, *p* = 0.1407), though higher categories showed trends toward increased odds of cognitive impairment. Similarly, atherosclerosis showed a non‐significant overall effect (χ^2^ = 5.73, df = 3, *p* = 0.125) but the highest level was associated with more than 9.7 times the odds of dementia (Severe vs none: OR = 9.73, 95% CI = 1.69–77.11, *p* = 0.022). None of the other neuropathological variables showed significant associations with odds of cognitive impairment, although many of the variables had low numbers of observations in certain cells.

**FIGURE 3:**
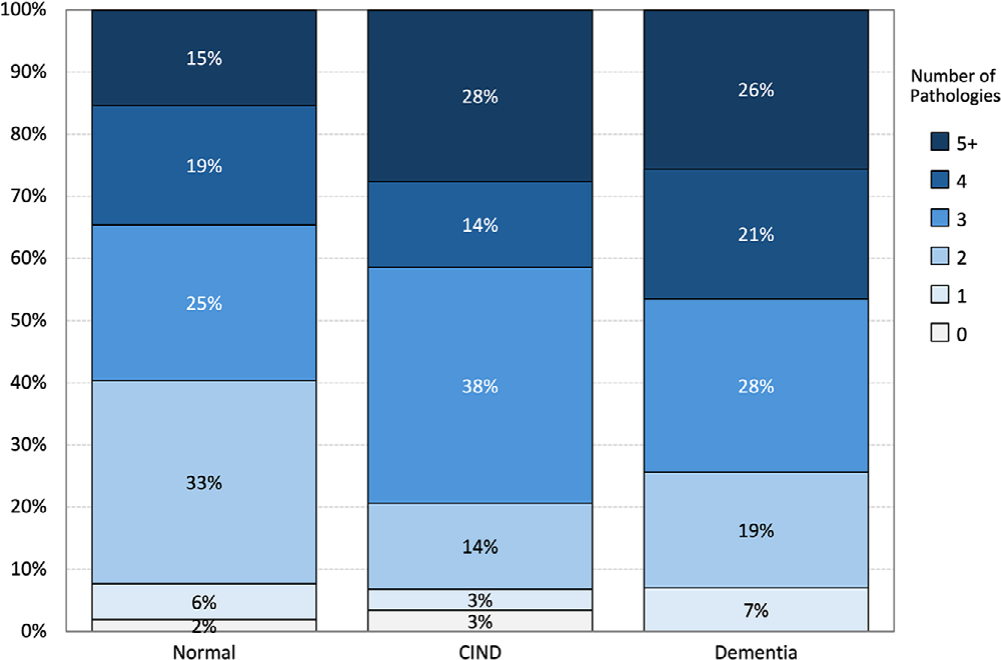
Graphs of the percentage (%) of cases that contained the number of pathologies (range of 0 to 7) by cognitive status (normal, CIND, dementia). The maximum total was nine pathologies (same as those listed in Figure 2) including: hippocampal sclerosis, ARTAG, CAA, atherosclerosis, arteriolosclerosis, old microinfarcts, TDP‐43, intermediate/high ADNC, and LBs (details of inclusion criteria within Figure 2 legend). No cases had more than seven of the listed pathologies. ADNC, Alzheimer disease neuropathologic change; ARTAG, aging‐related tau astrogliopathy; CAA, cerebral amyloid angiopathy; CIND, cognitively impaired no dementia; LBs, Lewy bodies.

When examining the total number of these pathologies in each of the 124 cases, only 2% of cases contained none (i.e. 0), with most cases having two to five pathologies (Figure 3). No individuals contained all nine pathologies, or eight pathologies; only 2% contained seven. The total number of pathologies based on cognitive status at last evaluation (Figure 3) was highest in the dementia group. For example, 26% of individuals in the dementia group had five or more pathologies compared to 15% in those in the normal cognition group. All individuals with dementia had at least one pathology. Among those with dementia, three individuals had only one pathology: one had old hemorrhagic infarcts, one had LB pathology‐ DLBD, and one had HS in addition to PSP. Given PSP was only identified in one individual it was not included in the co‐pathology count. It is important to note; there were no cases in the dementia group that did not have any pathologies. At the other end of the spectrum, 59% of participants with normal cognition had three or more neuropathological findings and only 2% had no pathologies (Figure 3). Furthermore, 33% of individuals with normal cognition had two pathologies compared to 19% of individuals with dementia.

## 4 DISCUSSION

The LifeAfter90 study sought to examine a demographically heterogeneous group of oldest‐old individuals to understand the presence of ADRD pathologies and determine their association to cognitive status. In this cohort, we reveal the most common pathologies to be those of AD albeit at low densities/distributions, and ARTAG. Many cases contained more than one pathology (Figure 2, Figure 3); consistent with prior literature.^10^ Furthermore, AD pathologies were significantly associated with cognitive impairment. Over 40% of participants were classified as cognitively normal at their final clinical evaluation, reinforcing prior observations substantial neuropathologic burden may be present in the absence of cognitive impairment/dementia, especially in the oldest‐old.^3^, ^50^

The current study examined individuals aged 90 and above from many demographic backgrounds lacking a dementia diagnosis in their electronic medical records at enrollment. This adds to a limited literature on neuropathology and its association with cognitive status in persons aged 90 and above. Previous US‐based studies examining cognition and neuropathology in later life had relatively small samples sizes^5^ perhaps reflecting the challenges in recruiting/evaluating this scarce population into longitudinal research programs. Samples have been highly selective, but in different ways. The 90+ Study, the Georgia Centenarian Study, and others that combined data from numerous individual studies have differences in inclusion/exclusion criteria with respect to cognitive/dementia status and demographic characteristics.^16^, ^17^, ^50^ For the 90+ Study, participants 99% identified as White, 30% had less than a high school education, 77% were women, and 41% had dementia at baseline.^4^ For the Georgia Centenarian study examining 49 decedents, 88% identified as Caucasian, 90% were women, 48% had less than a high school education, and individuals across the cognitive spectrum were included.^16^ Another study combining data from the Nun Study, the University of Kentucky Alzheimer's Disease Center, and the Georgia Centenarian study (*N* = 77, age range of 98‐107 years), had 89.6% women and many participants having cognitive impairment, indicated by average final MMSE score of 15.5/30.^17^ In the current cohort, 49% identified as White, 60.5% were women, and 25.4% had less than a high school education/general education development (GED) equivalent (Table 1). The current cohort is relatively small and has unique selection factors, including 17% identifying as Asian, 19% Latino, and 12% Black, in addition to all individuals being long‐term enrollees in a major health care provider organization. It is important to consider how study differences in demographics, cognitive status, selection factors, and recruitment can be associated with differences in neuropathology severity. For example, community‐based volunteers with clinical AD had significantly less severe AD pathology and more infarcts compared to clinic‐based persons.^51^ As more studies accumulate in the oldest‐old, it will aid in filtering out study specific differences to identifying common associations of neuropathology with cognitive status.

In this cohort, findings are largely consistent with previous cohorts of the oldest‐old in denoting AD pathologies are common^10^, ^12^, ^13^, ^17^. In LifeAfter90, 79% of participants had at least a low level of ADNC, with 21% classified as Not ADNC. Other studies had somewhat similar frequencies of AD pathologies within the oldest‐old.^5^, ^16^, ^17^ While the presence of some level of AD pathology is common, there are studies providing evidence of the relationship of AD pathologies to cognitive decline, with some denoting still unaccounted contributors.^14^, ^15^, ^52^, ^53^, ^54^, ^55^ With respect to Lewy pathology, 24% met criteria for BLBD, TLBD, or DLBD in the LifeAfter90 cohort; this was similar to estimates of 17‐22% in other oldest‐old cohorts.^5^, ^16^, ^17^ Examining the LB stages further, one study revealed TLBD in 9/77(∼12%) and DLBD in 4/77(5%); we had similar frequencies with ∼10% and 5% respectively.^17^

With vascular pathologies, some results differed from prior studies. Microinfarcts were identified in 22% of LifeAfter90 participants, lower than ∼50% reported in the 90+ Study,^11^ suggesting differences in cohort vascular health and/or other procedural/inclusion differences as 90+ included persons with dementia at baseline. In LifeAfter90, microinfarcts were not significantly associated with cognitive status; this contrasts with the 90+ Study.^11^ For CAA, 65% of cases had at least mild densities with no significant association with cognitive status. Previous studies, based on non–oldest‐old cohorts, indicate moderate‐to‐severe CAA associated with dementia due to its impact on cerebral blood flow and potential hemorrhagic damage.^56^ Within the oldest‐old CAA is common, being reported in 54% of the 90+ Study, and having an OR of dementia of 2.2.^5^ These differences may represent the variable contributions of cerebrovascular disease pathology across participant cohorts.^8^

TDP‐43 pathology was in nearly a quarter of cases and frequently co‐occurred with other pathologies. Previous studies, in younger old cohorts, shown certain types of TDP‐43 pathology, more recently referred to as LATE‐NC, are frequently seen alongside AD pathology and associated with cognitive decline in late life^9^, ^57^. Previous papers revealed a relationship of cognitive impairment to TDP‐43,^57^, ^58^, ^59^ while the current study did not. In the current study, there were changes in methodologies with TDP‐43 staining (Table S1). In a preliminary analysis, there was a greater presence of TDP‐43 deposits with the phosphorylated TDP‐43 antibody (35%) when compared to the unmodified TDP‐43 antibody (18%). However, there were no significant differences with respect to age at death, sex, race/ethnicity, or final cognitive diagnosis, based on TDP‐43 antibody (Table S2). Hippocampal sclerosis (HS) was rare (3%) with two of the four HS cases having dementia. There have been higher HS frequencies in the 90+ Study and Georgia Centenarian Study at 10‐11%^13^, ^16^, albeit numbers were low and there were differences in inclusion criteria‐ such as inclusion of persons with dementia at baseline.

Many individuals in the current study had multiple neuropathologies in alignment with literature.^5^, ^15^, ^16^, ^17^, ^50^ One study incorporating data from 77 cases, found 66% of cases had two more of: Braak NFT stage greater than II, CAA, arteriolosclerosis, HS, LBD, and/or large infarcts.^17^ Similar to published criteria^39^, oldest‐old cases with Lewy pathology in this study exhibit co‐occurring AD pathology; LBs rarely occur in isolation, and their clinical impact often depends on accompanying AD pathology.^39^ ARTAG was observed in over half of the cohort, agreeing with other reports identifying ARTAG as a age related and not strongly correlating with cognitive decline.^60^

A notable discrepancy between neuropathological burden and cognitive diagnosis in our study was observed among the 59% of participants with normal cognition who had three or more neuropathologies at autopsy (Figure 3). Furthermore, the distribution of vascular pathologies as well as Lewy body stage was not statistically significant across cognitive status groups, albeit numbers were low in some groups (Table 2). No cases within the normal cognition group were classified as high ADNC, with 28.8% as intermediate ADNC; 21% of cases with normal cognition had brainstem, transitional or diffuse LBs, and 22% had TDP‐43 deposits (Table 2). These individuals may represent cognitive resilience, the ability to maintain normal cognition despite significant neuropathological burden^61^, ^62^. In studies of the oldest‐old, resilience has been associated with the absence of HS, and lower burden of CVD, ARTAG, and/or TDP‐43 deposits^63^.

The LifeAfter90 study has some limitations. Autopsy participants were recruited exclusively from Kaiser Permanente Northern California having a predominantly urban catchment area; consented to longitudinal follow‐up and brain donation. This sample may not capture the full spectrum of demographics present across different healthcare systems, socioeconomic backgrounds, and/or rural regions. In the current cohort, the interval between a participants' final clinical evaluation and death was a mean of 0.6 years (range of 0–3.5 years), which may affect strength of associations. Although consistent protocols and current guidelines were used, sampling parameters may still differ across studies. While the study adhered to NIA‐AA and NACC guidelines for tissue processing, specific brain regions sampled such that rostral/caudal and medial/lateral extents of areas sampled may differ and only one hemisphere was evaluated.^35^ Furthermore, although numerous criteria exist for vascular abnormalities^36^, ^64^, ^65^, ^66^, ^67^, ^68^, there is no universally accepted guidelines, and many scales are subjective in nature and dependent on area evaluated. Lastly, although over 100 individuals were included, this study is still relatively small. AD neuropathologies were more prevalent so results for these may be more precise. Findings for less prevalent pathologies (i.e., HS) should be considered exploratory due to limited statistical power.

The LifeAfter90 study has several notable strengths, especially being one of the few neuropathological studies to recruit a demographically heterogenous cohort of oldest‐old individuals. Although the overall sample size is small, the proportions of participants across multiple race/ethnicities are the highest to date to our knowledge with respect to neuropathologic studies of people aged 90 and older, and we anticipate higher numbers in the future as the study progresses. Hence, although the current study is underpowered to denote statistical significance in differences by ethnoracial groups, continuation of this important study will provide further data for future analysis. There are other studies with autopsy components that draw from the Kaiser Health system focusing on enrollment at younger ages; these include the Kaiser Healthy Aging and Different Life Experiences Study (KHANDLE), which enrolls individuals aged 65 and older, and the Study of Healthy Aging in African Americans (STAR), which enrolls African Americans aged 50 and older^69^, ^70^, ^71^, ^72^, ^73^, ^74^. KHANDLE and STAR recently commenced brain donation and, in the future, will be wonderful comparison groups as they are embedded in the same population of an integrated healthcare delivery system as LifeAfter90. Its community‐based design, longitudinal clinical data, and rigorous blinded neuropathological assessments following NIA‐AA/National Alzheimer's Coordinating Center (NACC) guidelines further strengthen the validity of findings. Importantly, having individuals at baseline with no record of dementia diagnoses in their health records, followed longitudinally, and at death showed heterogeneity of clinical cognitive status, enables a comprehensive examination of the relationship between neuropathology and cognitive impairment in very late life.

These exploratory findings suggest AD pathologies are associated with dementia in this cohort of individuals 90 and older. Examination of other pathologies was limited due to lower prevalence resulting in small cell sizes. Nevertheless, the presence of additional pathologies‐such as TDP‐43, LBs, vascular disease, and ARTAG, highlights the complexity and heterogeneity of neurodegenerative processes in very late life. Given the number of individuals enrolled in brain donation in LifeAfter90, autopsy numbers are expected to triple in the next few years, giving the opportunity to evaluate neuropathological associations within a larger cohort. In future studies, we aim to investigate relationships of neuropathologies to midlife, survival, environmental exposures, and lifestyle behaviors data. These analyses will enhance our ability to identify modifiable risk and protective factors for late‐life cognitive impairment in the oldest‐old. Future studies investigating contributions to inclusion/exclusion in autopsy studies are valuable in understanding selection biases and barriers to participation. In summary, these initial neuropathological results from the LifeAfter90 study suggest while pathological burden is complex and varied, AD pathology remains an important contributor to cognitive impairment/dementia in very advanced age having important implications for ADRD therapy and treatment in the oldest‐old.

## CONFLICT OF INTEREST STATEMENT

The authors declare no conflict of interest. Author disclosures are available in the Supporting Information.

## CONSENT STATEMENT

Written informed consent was obtained for each participant and/or their legal authorized representative during life and IRB for Kaiser Permanente Northern California and University of California Davis (UCD) approved protocols were used. The study involved data from deceased individuals and is not considered human subjects. The study was performed in accordance with the ethical standards as laid down in the 1964 Declaration of Helsinki and its later amendments or comparable ethical standards.

## Supporting information




Supporting Information

Supporting Information
