# Supplementary material for: Neuropathology in a diverse cohort of oldest‐old: The LifeAfter90 study
Source: Alzheimers Dement. 2026 Jul 24;22(7):e71634. doi: 10.1002/alz.71634 (PMC13400840; doi:10.1002/alz.71634)
Supplement: Supplementary file 1 — Supporting Information [file ALZ-22-e71634-s002.docx]

Supplemental table 1. Staining details

| **Antibody** | Aβ (Amyloid Beta)* | AT8 (Tau) | TDP-43/pTDP-43* | LB509 (α-Synuclein) |
| --- | --- | --- | --- | --- |
| **Antigen/**  **Epitope** | Amino acids 18-22 of beta Amyloid (VFFAE) | Phosphatase-sensitive epitope on PHF-Tau | Recombinant protein and cell lysate at TAR DNA binding protein / Human TDP-43 phosphorylated on serines 409 and 410 | α-Synuclein |
| **Reactivity** | Monoclonal | Monoclonal | Monoclonal | Monoclonal |
| **Species** | Human, mouse | Human | Human, mouse, rat | Human |
| **Vendor** | Covance/Biolegend | Invitrogen | Novus Biologicals /Cosmo Bio USA | Invitrogen |
| **Catalog #** | SIG-39200 /800708 | MN1020 | H00023435-M01 / CAC-TIP-PTD-M01A | 18-0215 |
| **Regions stained** | Superior and middle temporal gyri  Posterior hippocampus  Striatum at level of anterior commissure with nucleus basalis of Meynert  Substantia nigra  Olfactory bulb and tract  Middle frontal gyrus  Cerebellum with dentate nucleus | Superior and middle temporal gyri  Visual cortex  Posterior and anterior hippocampus  Amygdala  Striatum at level of anterior commissure with nucleus basalis of Meynert  Substantia nigra  Olfactory bulb and tract  Middle frontal gyrus  Cerebellum with dentate nucleus | Superior frontal gyrus  Posterior hippocampus  Amygdala  Inferior temporal gyrus | Cingulate gyrus  Amygdala  Substantia nigra  Olfactory bulb and tract  Medulla  Middle frontal gyrus |

*In December 2023 the Cosmo Bio pTDP-43 antibody replaced the Novus TDP-43 antibody and the Aβ Covance antibody was replaced with the Biolegend.

Supplemental Table 2. Characteristics of Participants By Type of TDP-43 Antibody

|  | **TDP-43 antibody** | |  | |
| --- | --- | --- | --- | --- |
|  | **non-Phospho-TDP (N = 77)** | **Phospho-TDP (N = 43)** | **Total* (N = 120)** | **P Value** |
| **Age at Death, years** | | | | |
| Mean (Range) | 95.7 (90.5 - 105.7) | 96.3 (93.2 - 102.4) | 95.9 (90.5 – 105.7) | 0.23 |
| **Sex** ^a^ | | | | |
| Male | 35 (45.5%) | 13 (30.2%) | 48 (40.0%) | 0.12 |
| Female | 42 (54.5%) | 30 (69.8%) | 72 (60.0%) |  |
| **Race/Ethnicity** ^a^ | | | | |
| Asian | 14 (18.2%) | 7 (16.3%) | 21 (17.5%) | 0.89 |
| Black | 8 (10.4%) | 7 (16.3%) | 15 (12.5%) |  |
| Latino | 14 (18.2%) | 9 (20.9%) | 23 (19.2%) |  |
| Multiple/Other/missing | 2 (2.6%) | 1 (2.3%) | 3 (2.5%) |  |
| White | 39 (50.6%) | 19 (44.2%) | 58 (48.3%) |  |
| **Cognitive Status at Last Evaluation** ^a^ | | | | |
| Normal | 33 (42.9%) | 17 (39.5%) | 50 (41.7%) | 0.92 |
| CIND | 18 (23.4%) | 10 (23.3%) | 28 (23.3%) |  |
| Dementia | 26 (33.8%) | 16 (37.2%) | 42 (35.0%) |  |
| **LATE-NC** ^a *^ | | | | |
| None | 63 (81.8%) | 28 (65.1%) | 91 (75.8%) | 0.048* |
| Stages 1-3 | 14 (18.2%) | 15 (34.9%) | 29 (24.2%) |  |

**Note:**

Age at death was evaluated between groups via t-test, other variables used ^a^ Fisher exact for p-value *4 cases had insufficient tissues/missing and were unable to assess for TDP
